# Molecular and cytogenetic analyses of a patient with Prader-Willi syndrome who also had the phenotype of Angelman syndrome

**DOI:** 10.1186/1687-9856-2013-S1-P56

**Published:** 2013-10-03

**Authors:** Kenichi Miyako, Atsuko Kawano, Yuichi Mushimoto

**Affiliations:** 1 Fukuoka Children's Hospital, Japan

## 

Most cases of Prader-Willi syndrome are caused by partial deletion of the paternally derived chromosome 15, while maternally derived chromosome 15 is responsible for Angelman syndrome. We report the results of molecular and cytogenetic analyses of a patient who was given a final diagnosis of Prader-Willi syndrome, but also had manifestations consistent with Angelman syndrome.

The patient was a 15-year-old boy. After birth, Prader-Willi syndrome was diagnosedon fluorescence in situ hybridization (FISH), performed because of muscular hypotonia, failure to thrive, and bilateral cryptorchidism. However, at the age of 2 years, the diagnosis was revised to Angelman syndrome because of atypical absence, characteristic electroencephalographic discharges, mental retardation, and excessive laughter. At the age of 14 years, type 2 diabetes developed, and he is now receivinginsulin glargine and voglibose. The height is 147.0 cm (-3.68 SD), and the body weight is 55.0 kg (body mass index, 25.5 kg/m^2^). He cannot speak any meaningful words or walk. He has hyperphagia, hypopigmentation, almond-shaped eyes, small hands and feet, and a large mouth and jaw.

Chromosomal examination by G-banding stain revealed that the karyotype was a mosaic composed of 45,XY,der(1)t(1;15)(p36.3;q13),-15 and 46,XY,der(1)t(1;15) (p36.3;q13),-15 ,+mar. FISH did not detect signals of UBE3A/D15S10 with the probe for Angelman syndrome or SNRPN for Prader-Willi syndrome on the der(1) chromosome. The marker chromosome was derived from the short arm of chromosome 15. Methylation-specific PCR amplified the SNRPN gene using only primers specific for methylated gene. FISH for 1p36 deletion syndrome did not detect the deletion on the der(1) chromosome.

We concluded that his diagnosis was Prader-Willi syndrome caused by the partial deletion of the long arm of chromosome 15, which had translocated onto chromosome 1. The clinical manifestations might have beenmodified by the complicatedstructural changes in chromosomesas well as by possible terminal deletion of the short arm of chromosome 1, which could not be detected on FISH.

